# Effects From Dietary Addition of *Sargassum* sp., *Spirulina* sp., or *Gracilaria* sp. Powder on Immune Status in Broiler Chickens

**DOI:** 10.3389/fvets.2022.928235

**Published:** 2022-06-13

**Authors:** Hanan S. Al-Khalaifah, A. Al-Nasser, T. Surrayai

**Affiliations:** Environment and Life Sciences Research Center, Kuwait Institute for Scientific Research, Kuwait City, Kuwait

**Keywords:** algae, immune response, poultry, *Sargassum* sp., *Spirulina* sp., *Gracilaria* sp.

## Abstract

Algae are innovative and significant nutrient sources with various health benefits when used as additives in animal feed. The study aims to examine the effect of different inclusions of three algae species, *Sargassum* sp., *Spirulina* sp., and *Gracilaria* sp. on the immune response of broiler chickens, as measured by the cellular immune response, humoral immune response, intestinal microbial counts, hindgut acidosis, and hematological measures. Here is a list of the seven experimental treatments (TRT). TRT 1 was the control group without algae; TRT 2 was supplemented with *Sargassum* sp. at 1% of the diet; TRT 3 with *Sargassum* sp. at 2% of the diet; TRT 4 with *Spirulina* sp. at 5% of the diet; TRT 5 with *Spirulina* sp. at 7.5% of the diet; TRT 6 with *Gracilaria* sp. at 0.5% of the diet; and TRT 7 *Gracilaria* sp. at 1% of the diet. Each treatment involved five replicates with 17 broiler chickens each, and the analyses were triplicated. The results showed that including algae in the feed ration of broiler chickens induces a higher cellular response than the control group, represented by T-cell response in the wattle area (*P* = 0.037). *Sargassum* sp. at 1 and 2% enhanced IgA antibody titers significantly and *Gracilaria* sp. at 5% enhanced IgY antibody titers, *P* = 0.045 and *P* = 0.030, respectively. All algal inclusions inhibited the growth of *Salmonella* sp. and improved LAB counts in the intestine of broilers, excepting the *Gracilaria* sp. at 0.5%, where LAB counts were similar to the control group. The *E. coli* counts decreased numerically but not significantly. Blood lymphocytes were enhanced while white blood cells (WBC) and heterophils were decreased as a results of algal inclusions. In conclusion, supplementing broiler chickens with algae could enhance their cellular and humoral immune status and promote healthy microflora in their guts.

## Introduction

Broiler performance can be negatively affected by harsh environmental conditions, such as heat stress. This stress results in the partitioning of nutrients away from growth and moving toward the physiological process associated with heat stress resistance. This will lead to decreased production and profit for commercial poultry companies, especially during global crisis such the corona virus crisis (1–3). There has been some interest in supplementing poultry with effective feed ingredients that improve production performance parameters in recent years. One such ingredient is marine algae. Dietary supplementation with marine algae is claimed to serve several essential functions in the body, such as providing a source of metabolic energy, improving production performance, and acting as precursors for eicosanoids production (4, 5). Furthermore, it is well-known that using antibiotics as growth promoters in food animals is increasingly being banned in most parts of the world due to the development of antibiotic resistance pathogenic organisms, which compromise human and animal health. Therefore, marine algae as feed supplements could be a solution for problems related to antibiotics in feed. *Sargassum* sp., *Spirulina* sp., and *Gracilaria* sp. are some examples of marine algae used in poultry feed (6–11).

*Sargassum* sp. is a naturally occurring brown alga that has been extensively used in various industries and for animal nutrition (12). It is normally found in shallow waters and coral reefs. There are also some cases of floating populations of these algae. It is believed to impact the performance and health of animals, including the productive performance of chickens (13). These species also claimed to increase the fat content in broilers by reducing cholesterol in the produced meat, eggs, and their sera (14, 15). Additionally, the nutritive value of *Sargassum* species and their utilization as feed have been described in numerous studies. They are enriched with minerals, vitamins, essential amino acids, polysaccharides, omega-3 and omega-6 fatty acids, and sterols (15–17). Also, Nadal et al. (18) stated the presence of antibiotic substances in the extracts of these algae.

*Spirulina* sp. is a microscopic spiral-shaped blue-green alga living in sea and freshwater. Dried *Spirulina* comprises around 60% (51–71%) protein, with all essential amino acids. Its lipid content is about 7% by weight (19). Similarly, it has a wide range of vitamins, including vitamins B_1_ (thiamine), B_2_ (riboflavin), B_3_ (nicotinamide), B_6_ (pyridoxine), B_9_ (folic acid), vitamin C, vitamin A, and vitamin E (19). Additionally, it is a good source of potassium, calcium, chromium, copper, iron, magnesium, manganese, phosphorus, selenium, sodium, and zinc (19). It acts as an immunostimulant and has anticarcinogenic and antiviral properties (20). Mirzaie et al. (21) reported decreased concentration of stress hormone and enhanced humoral immune response, and elevated antioxidant status in the groups supplemented with *Spirulina* compared to the control group. Interestingly, Abdel-Moneim et al. (22) reported that *Spirulina* sp., in combination with selenium nanoparticles, enhanced the antioxidant status and immune function of broiler chickens significantly.

Red macroalgae such as *Gracilaria* sp. are enriched in protein and used in a dried form as protein sources in formulated animal feed (23). It is a suitable applicant for intensive culture because of its ability to reach higher yields and economically valuable products in aquaculture. A study was conducted to evaluate the effects of a diet containing *Gracilaria* sp. waste on duck's lipid profiles, including cholesterol, triglycerides, low-density lipoprotein (LDL), and high-density lipoprotein (HDL). A diet supplemented with 12.5% *Gracilaria* waste significantly affected the blood lipid profiles of the ducks, lowered triglycerides and LDL cholesterol, and increased blood HDL levels (24).

Marine algae have been well documented in the literature for their anti-inflammatory properties. E.g., Wu et al. (25) studied the anti-inflammatory activities of polysaccharides from three algae species, *viz., Porphyra tenera* (red algae), *Monostroma nitidum* (green algae), and *Sargassum cristaefolium* (brown algae), by evaluating the inhibition of nitric oxide (NO) production in murine macrophages. The authors determined that marine algae possess an anti-inflammatory effect by inhibiting the signaling pathways of NO production.

In the current study, we aim to investigate the effect of different inclusions of *Sargassum* sp., *Spirulina* sp., and *Gracilaria* sp. on the cellular immune response, humoral immune response, and intestinal microbial counts, hindgut acidosis, and hematological measurements in broiler chickens. The hypothesis is that the algal inclusions will improve the aforementioned parameters, but the different effects of the different concentrations should be emphasized. Although some studies have investigated the effect of algae on the productive performance parameters in broiler chickens, there is relatively limited data on the direct effects of the different kinds and levels of algae used in this study on the parameters mentioned above. Accordingly, this study is innovative and its results are important to improve the quality of poultry industry and to reduce the cost of feed rations that contribute to about 75% of the total operation cost of poultry.

## Materials and Methods

### Animal Welfare, Housing, and Diets

This research was approved by the Department Committee of the Environment and Life Sciences Research Center at Kuwait Institute for Scientific Research under Project No. FA127C (2017). These procedures and protocols adhered to the official animal welfare guidelines and regulations encoded with Reference No. PMO/PV/GM/073/2015. According to this protocol, experimental animals were treated humanely without pain, stress, or harm.

In this study, 1-day-old male Cobb 500 broiler chicks were used. Both water and feed were provided *ad libitum*. The broiler chickens were raised in cages rather than floor pens to reduce operation costs and to improve space utilization. The broiler chicks were fed a starter diet from hatch until 7 days (1 week) of age, a grower diet from 8 to 21 d (2–3 weeks) of age, and a finisher diet from 22 to 35 days (4–5 weeks) of age. The feed rations were formulated as per Cobb 500 guidelines with corn and soya (26). The chickens were fed the experimental diet from 1 day till slaughter at 35 days of age.

A total of 595 broilers were used in the experiment, distributed into seven batteries, 85 birds per battery. Each battery included five levels, and the space of each level was 0.85 m^2^. There were 17 birds on each level, occupying a space of 0.05 m^2^ each. There were seven experimental treatments (TRT), like following: TRT 1, the control group, was given a soybean basal diet without added algae; birds in TRT 2 received the same basal diet as TRT 1 birds and supplemented with *Sargassum* sp. at 1% of the diet; birds in TRT 3 received the same basal diet as TRT 1 birds and supplemented with *Sargassum* sp. at 2% of the diet; birds in TRT 4 received the same basal diet as TRT 1 birds and supplemented with *Spirulina* sp. at 5% of the diet; birds in TRT 5 received the same basal diet as TRT 1 birds and supplemented with *Spirulina* sp. at 7.5% of the diet; birds in TRT 6 received the same basal diet as TRT 1 birds and supplemented with *Gracilaria* sp. at 0.5% of the diet, and birds in TRT 7 received the same basal diet as TRT 1 birds and supplemented with *Gracilaria* sp. at 1% of the diet. Based on the results of the proximate analyses of the algae, these levels of algae were chosen. The diets were performed as isonitrogenic and isocaloric diets between study groups. The 1-day-old chicks were provided with 24 h of light during the first 3 days of the brooding period to ensure that they had enough time to discover the feed and water. Subsequently, a step-down lighting program was followed. Artificial bulbs were used as the light source.

### Sample Collection

During weeks 3 and 5 of age, blood samples were collected from the branchial veins of birds in vacutainer tubes (K2EDTA). In each tube, 8–10 ml of blood was collected from five chickens from each treatment. Similarly, ceca samples were collected and analyzed for microbial populations. Triplicate analyses were performed. Birds were selected randomly for each analysis.

### Cellular Immune Response

Phytohemagglutinin (PHA) was dissolved in pyrogen-free phosphate-buffered saline (PBS) and was injected into the subcutaneous layer of the broiler skin. The subsequent swelling at the injection site (wattle) was measured after 24–72 h, which was considered an index of cell-mediated immunocompetence. According to Martínez-Galero et al. (20) and Mirzaie et al. (21), ten chickens were used in this study at the age of 5 weeks. The injection site was marked before injection, and a micrometer was used to measure its thickness. Hereafter, the birds were injected intradermally in the wattle with 0.5 mg of PHA-P in 0.1 ml of PBS. The thickness was typically measured at 24 h after injection, yet 24 h did not reflect the peak of the reaction; it could be measured (to the nearest 0.01 mm) at 0, 24, 48, and 72 h after PHA-P injection. Wattle swelling was calculated as the difference between the prior and post-injection thicknesses of the wattle.

### Humoral Immune Response

Ten broiler chickens of 5 weeks of age from each treatment were tested for humoral immune response. Antibody titers were assessed using sheep RBC. The broiler chickens were injected with 1 ml of diluted sheep RBC solution (7% v/v in 0.9% NaCl). Blood serum samples were collected after a week of injection using centrifugation methods, and differential antibody titers were measured using commercial ELISA kits. Each well in the 96-well tray was filled with 50 L of the respective standards. Then, 40 μl of each sample was added to the sample wells, and afterward, 10 μl of biotin-conjugated anti-chicken antibody. Then, 50 μl of streptavidin-HRP was carefully added to each sample avoiding the blank control wells, and reagents were thoroughly mixed. The plate was covered with a sealer and incubated at 37°C for 60 min. The sealer was detached after incubation, and the plate was washed with wash buffer five times; the wells were overfilled and soaked for at least 30 s to 1 min (27, 28). Paper towels were used after each washing to blot the plates. Then 50 μl of substrate solution A was added to each well, followed by 50 μl of substrate solution B (light-sensitive substrate solution B should not be exposed to light). The plate was sealed with another sealer and incubated in the dark for 10 min at 37°C. Simultaneously, after adding 50 μl of stop solution to each well, the blue solution immediately turned yellow. Lastly, the optical density (OD) value was measured at 40 nm within 30 min after adding a stop solution using a microplate reader (27).

### Microbial Counts in the Chicken's Gut

Lactic acid bacteria (LAB), *Escherichia coli* (*E. coli*), and *Salmonella* were analyzed by extracting the cecum substance as defined by Schoeni and Doyle (29). For each treatment, ten chicken samples aged 35 days were used. All chicken samples were slaughtered on the farm and transferred to the laboratory for further analysis under refrigerated conditions. The collected chicken samples were prepared in the lab according to the protocol by Al-Khalaifa et al. (30). First, the chickens were weighed and washed with 1:2 diluted disinfectant. The abdominal area was de-feathered and sterilized with 70% ethanol before dissecting. Then, the skin was cut with a sterile scissor and removed from the abdomen area using sterile forceps. The covering membrane was carefully cut to reach the chicken's digestive system. The lower intestine was surgically exposed, the caeca were aseptically removed, and their weights were recorded. LAB, *Salmonella*, and *E. coli* were isolated from the caeca by extracting its contents according to Schoeni and Doyle (29). Each caecum's content was squeezed into a sterile petri dish and then split lengthwise with a sterile scalpel and rinsed with 0.85% (w/v) NaCl sterile solution (1:9 v/v) to remove the content. Any residual cecal content was gently removed by scraping the cecal epithelium. The crude extract of the caeca was transferred to a sterile stomacher bag and homogenized for 3 min.

The collected crude extracts were directly used for microbial analysis. LAB, *E. coli*, and *Salmonella* counts were determined using standard microbiological methods by plating serial dilutions to respective agar plates (27, 28). Samples were then analyzed using spreading technology. *E. coli* and *Salmonella* count experiments were accomplished using Brilliance *E. coli* selective and Xylose-Lysine-Desoxycholate agar media (Oxoid), respectively, whereas LAB experiments were carried out using de Man, Rogosa, Sharpe (MRS) media (Oxoid). From the crude samples, serial dilutions were prepared, and 0.1 ml of the prepared sample was spread onto the surface of the media with a sterile spreader. The plates were then incubated aerobically for 24 h at 37°C for both *E. coli* and *Salmonella*, while for LAB, the plates were incubated anaerobically for 48 h at 30°C. At the end of the incubation period, colonies were counted. These counts were transformed into log values.

### Hindgut Acidosis

Hindgut acidosis denotes cecum and/or colon acidity. The pH of the control and experimental treatment groups was taken to indicate broilers' health and their ability to resist pathogens. The test was conducted on broilers of 3 and 5 weeks, and ten broiler chickens were used for each treatment. Hindgut digesta was collected into tubes, and the pH value was measured with a probe.

### Hematological Measurements

In this procedure, computerized hemocytometers were used to evaluate and count immune and red blood cells (RBC). Blood samples (about 8–10 ml per tube) were collected from the brachial veins of the chicken in vacutainer tubes (K2EDTA). The samples were stored in an icebox and were rapidly analyzed. Total and differential blood quality parameters like hemoglobin (HGB), RBC, white blood cells (WBCs), mean corpuscular hemoglobin concentration (MCHC), hematocrit (HCT), red cell distribution width (RDW), mean corpuscular volume (MCV), thrombocyte platelet count (PLT), and mean corpuscular hemoglobin (MCH) were measured using 3,500 hematocytometer (Abbott Laboratories, Abbott Park, IL, United States). Ten broilers per treatment were used.

### Statistical Analysis

A total of seven experimental treatments were used. In each treatment, 340 birds were randomly housed in four multi-floor batteries with five levels each, each level is considered as a replicate, *n* = 5. Each level had 17 birds for 85 birds in the battery. The levels were considered as replicates (five replicates per treatment). We compared the effects of the dietary treatments using one-way ANOVAs *via* the general linear model procedure in Minitab. Differences among the treatments were considered to be statistically different at *P* ≤ 0.05. Prior to analysis, the data were arcsine transformed to improve normality. Pairwise Tukey *post hoc* comparisons were performed to identify significant differences between groups.

## Results

### Cellular Immune Response

Wattle swelling changes as affected by the different dietary algal inclusions (Table 1). In Table 1, the results revealed that using algae in the feed rations of broiler chickens induces a higher cellular response than the control group, represented by T-cell response in the wattle area (*P* = 0.037).

**Table 1:** Wattle swelling changes, antibody titers, and microbial count as affected by different algal inclusions in 5-week-old broiler chickens.

	**Wattle swelling (mm)**	**Chicken antibody concentration (ng/ml)**			**Microbial count (CFU/g of Caeca)**			
		**IgA**	**IgM**	**IgY**	**Log. LAB**	**Log. *E. coli***	**Log. *salmonella***	**Intestinal pH**
Control	1.47^b^	0.00^b^	0.03	0.09^b^	3.15^b^	7.15	1.25	7.24^a^
*Sargassum* sp. (1%)	1.65^a^	0.05^a^	0.03	0.11^b^	4.35^a^	6.40	No growth	7.23^a^
*Sargassum* sp. (2%)	1.63^a^	0.05^a^	0.02	0.07^b^	3.65^a^	6.50	No growth	6.12^b^
*Spirulina* sp. (5%)	1.80^a^	0.01^b^	0.03	0.10^b^	3.95^a^	6.65	No growth	6.54^b^
*Spirulina* sp. (7.5%)	1.78^a^	0.01^b^	0.03	0.08^b^	3.90^a^	6.40	No growth	7.01^a^
*Gracilaria* sp. (0.5%)	1.75^a^	0.01^b^	0.02	0.36^a^	3.15^b^	6.00	No growth	6.01^b^
*Gracilaria* sp. (1%)	1.64^a^	0.00^b^	0.03	0.09^b^	3.50^a^	6.05	No growth	6.50^b^
SEM	0.401	0.02	0.03	0.18	0.19	0.47	0.41	0.87
*P*-value	0.037	0.030	0.354	0.045	0.012	0.296	0.495	0.045

*^a, b^Means in column with no common superscripts are significantly different (p ≤ 0.05).*

*Values are expressed as means (n = 5 for each dietary treatment), pooled standard error of means (SEM).*

*IgA, Immunoglobulin A; IgM, Immunoglobulin M; IgY, Immunoglobulin Y*.

### Humoral Immune Response

Table 1 shows the effect of different levels of dietary algal inclusions on chicken antibody titers (i.e., IgA, IgM, and IgY) in 5-week-old broilers. It was found that *Sargassum* sp. at 1 and 2% significantly enhanced IgA antibody titers than the control group and the other dietary groups (*P* = 0.030) (Table 1). All other algal inclusions had no significant effect on IgA antibody titers. All dietary treatments had no significant effect on the IgM titer in broiler chickens. Table 1 also showed that IgY antibody titers were significantly enhanced after supplementing with *Gracilaria* sp. at 5%, compared to the control group and the other dietary groups (*P* = 0.045).

### Microbial Counts in the Chicken's Gut

fTable 1 represents the effect of different dietary algal inclusions on the microbial count in 5-week-old broiler chickens. According to Table 1, including *Sargassum* sp., *Spirulina* sp., and *Gracilaria* sp. at all concentrations in broiler feed rations inhibited the growth of *Salmonella* sp. in the intestine of broiler chickens. LAB counts were significantly elevated as a result of using all the algal inclusions, with the exception of *Gracilaria* sp. at 0.5%, where LAB counts were similar to the control group. The *E. coli* counts were decreased numerically in broilers fed with *Sargassum* sp., *Gracilaria* sp., and *Spirulina* sp., compared to the control diet, but this decrease failed to reach significance.

### Hindgut Acidosis

Table 1 shows the hindgut acidosis (i.e., intestinal pH value) of the 5-week-old broilers fed different algal inclusions. In Table 1, it was shown that inclusions of *Sargassum* sp. at 2%, *Spirulina* sp. at 5%, *Gracilaria* sp. at 0.5, and 1% significantly decreased the pH value of the intestine and increased its acidity (*P* = 0.045).

### Hemocytometry Analysis of Blood Samples

Table 2 presents the hemocytometry analysis of the blood samples of broilers fed the different dietary treatments. Based on Table 2, it is found that there is a significant effect of numerous dietary algal treatments on the blood hematological and biochemical traits in 5-week-old broilers for WBCs, heterophils, lymphocytes, monocytes, eosinophils, and basophils. Compared to the control group, broiler chickens supplemented with different algal inclusions at all levels significantly reduced their total WBC (*P* = 0.012). Birds fed *Spirulina* sp. at 5% of the diet displayed the lowest WBC among all the other dietary groups. Supplementing broiler chickens with all the algal inclusions significantly decreased the heterophils % (*P* = 0.017), excepting *Sargassum* sp. at 1% that has the same effect as the control diet. Lymphocytes % was improved after complementing the broiler diet with all the algal inclusions (*P* = 0.002), except *Sargassum* at 1%, which has the same effect as the control diet. Outcomes of Table 2 also demonstrate that monocytes % of the group supplemented with *Sargassum* sp. at 1% were the highest among the other dietary groups (*P* < 0.001). Furthermore, supplementing the broiler diet with *Spirulina* sp. at 7.5% resulted in the highest eosinophils % among all the other dietary groups (*P* = 0.016). *Spirulina* sp. at 7.5% and *Gracilaria* sp. at both 0.5 and 1% significantly increased the basophils % in broiler diets compared to the control group and other dietary groups. As shown in Table 2, the different algal inclusions did not affect the other blood biochemical parameters.

**Table 2:** Hematological and biochemical parameters of 5-week-old broilers fed different levels of dietary algae.

	**Treatment (g/kg)**								
**Parameter**	**Control**	***Sargassum***	***Sargassum***	***Spirulina***	***Spirulina***	***Gracilaria***	***Gracilaria***	**SEM**	***P*-value**
		**sp. (1%)**	**sp. (2%)**	**sp. (5%)**	**sp. (7.5%)**	**sp. (0.5%)**	**sp. (1%)**		
WBC (K/ul)	99.35^a^	90.05^b^	94.45^b^	85.45^c^	89.30^b^	90.56^b^	94.59^b^	2.52	0.012
Heterophils (%)	10.05^a^	11.20^a^	3.05^b^	5.48^b^	6.22^b^	5.23^b^	6.01^b^	2.13	0.017
Lymphocytes (%)	0.85^b^	1.17^b^	47.90^a^	31.30^a^	25.10^a^	36.23^a^	38.63^a^	4.34	0.002
Monocytes (%)	21.50^b^	47.50^a^	25.20^b^	22.45^b^	23.20^b^	20.25^b^	21.32^b^	0.66	<0.001
Eosinophils (%)	0.010^ab^	0.00^b^	0.006^b^	0.012^ab^	0.022^a^	0.00^b^	0.005^b^	0.003	0.016
Basophils (%)	38.05^b^	39.15^b^	23.85^b^	39.25^b^	54.45^a^	48.54^a^	50.10^a^	2.71	0.005
RBC (M/ul)	2.71	2.71	2.68	3.20	2.66	3.11	2.9	0.36	0.802
HGB (g/dl)	13.15	13.00	12.60	16.00	13.45	14.12	13.99	1.93	0.751
HCT%	31.20	30.05	31.80	38.00	31.30	32.10	31.78	5.32	0.834
MCV (fL)	115.00	111.50	116.00	118.00	117.00	115.98	117.10	5.43	0.913
MCH (pg)	48.40	47.55	46.45	49.80	50.50	50.12	49.51	1.70	0.510
MCHC (g/dl)	42.05	42,.80	40.10	42.10	42.90	42.05	42.00	0.94	0.346
RDW%	14.75	15.20	14.15	13.45	13.25	14.20	13.99	0.80	0.449
Thrombocytes/PLT (K/ul)	3.2	2.63	1.75	1.84	0.57	1.50	0.98	0.76	0.286

*Means within rows are significantly different at p ≤ 0.05, n = 5.*

*SEM, Standard error of the mean, calculated by one-way analysis of variance (ANOVA) and the general linear model procedure of Minitab; WBC, white blood cells; RBC, red blood cells; HGB, hemoglobin; HCT, hematocrit; MCV, mean corpuscular volume; MCH, mean corpuscular hemoglobin; MCHC, mean corpuscular hemoglobin concentration; RDW, red cell distribution width; PLT thrombocytes.*

*^a, b^Means in a column with no common superscripts are significantly different (p ≤ 0.05)*.

## Discussion

This study was designed to evaluate the effects of different inclusions of *Sargassum* sp., *Spirulina* sp., and *Gracilaria* sp. on the cellular immune response, the humoral immune response, intestinal microbial counts, hindgut acidosis, and hematological measurements in broiler chickens. Various selected levels of *Sargassum* sp., *Spirulina* sp., and *Gracilaria* sp. were used. These levels were selected based on the algae proximate analysis during formulation of the broiler feed rations.

All the dietary algal inclusions used in the present study induced a higher cellular response than the control group, represented by the wattle swelling changes. This shows the immunomodulation effect of algae on the specific cellular response of broilers, represented by T-cell activity. This response is essential for the immune system of birds.

This study shows that broilers supplemented with *Sargassum* sp. had significantly higher IgA antibody titers results than the other dietary groups, and broilers supplemented with *Gracilaria* sp. showed significantly higher IgY antibody titers than the other dietary groups. These results agree with those of Choi et al. (31), who stated that using brown algae in broiler feed rations significantly improved IgA and IgM concentrations in serum compared to the control group. Additionally, Kulshreshtha et al. (32) described that supplementing broiler diets with red algae enhanced the antibody titers in chickens. Changes in serum antibody titers are an accurate and direct indication of the status of the humoral immunity. Red seaweeds have been shown to control the humoral immune response and microbiota in birds (33). Similarly, brown algae are rich in minerals and organic acids, such as alginate, which has a stimulatory effect on cytokine secretion and, therefore, has a positive effect on the immune system (31). The exact mechanism underlying the effects of seaweed dietary supplements such as *Sargassum* and *Gracilaria* on the immune status of chicken is still unclear.

Seaweeds are one of the many materials that poultry can consume as an important source of bioactive compounds and contribute to their health by providing a variety of compounds. Seaweed extracts have antimicrobial and antiviral properties, as well as immunomodulatory effects (34, 35). Seaweeds could also be utilized as prebiotics to increase the productivity and health of poultry species. *Spirulina* is a kind of cyanobacterium (10). Recently, some studies on poultry have found that feeding *Spirulina* improves immunological functioning, which results in enhanced disease resistance, improved survival, and increased growth rates (3).

Interestingly, one study pointed out that *Salmonella* settlement in the excreta and ceca was limited by dietary supplementation of red seaweed (*Chondrus crispus*), which could be due to an increase in the development of *Lactobacillus* and a rise in the level of short-chain fatty acids (32). According to the same authors, the presence of more IgA in birds fed diets supplemented with *Chondrus crispus* confirmed the importance of macroalgae in immune system maturation. Bai et al. (36) found that Broilers' immune systems could be boosted by supplementing *Laminaria japonica* powder and antibacterial peptide (cecropin) in their diet. A feed containing 3% *L. japonica* powder and 300 mg/kg cecropin increased the serum Newcastle disease antibody titers and lymphocytes during the fattening period of broilers. *Laminaria japonica's* bioactive chemicals activated lymphocytes and altered their cell structure, affecting immunity (34).

In newly hatched chicks in commercial hatcheries, the concentration of volatile fatty acid and pH are insufficient to chemically suppress pathogens (37–39). Therefore, the supplementation of chickens with immunostimulants is essential to keep the health status of the flock (40). Once a chicken hatches into an environment heavily contaminated with bacteria, viruses, and protozoans, it must immediately begin to develop protective gut microflora. Under normal conditions, a 3–5 week period is required to develop a stable population of gut-associated bacteria, and their greatest numbers reside in the ceca. An anaerobic environment develops in the ceca that supports the growth of organisms such as *Bifidobacterium* spp. and *Bacteriodes* spp. Along with these bacteria, other LAB create a micro-ecology, which can be characterized by an acidic pH resulting from the production of undissociated volatile fatty acids in the caeca (acetic, butyric, propionic, and lactic acids) and antimicrobial substances that efficiently eliminate or kill many different pathogens. The effect of undissociated short-chain volatile fatty acids is well documented in numerous studies. They possess a high concentration with acidic pH and exert an antimicrobial effect. These acids are lipophilic, penetrating the bacterial cell wall and producing H^+^ ions, which in turn destroy the internal physiology of the bacterial cell (41).

The results confirmed that *Sargassum, Spirulina*, and *Gracilaria* inhibited the growth of *Salmonella* sp. and promoted beneficial intestinal flora such as *Lactobacillus* (i.e., LAB). These findings are in agreement with those of Wakwak et al. (42), Kulshreshtha et al. (43), and Fathi (44). *E. coli* counts were numerically lower when broilers were fed *Sargassum* sp., *Gracilaria* sp., and *Spirulina* sp. than when they were fed the control diet, but the difference was not statistically significant.

The enhanced acidity of the intestine indicates enhanced intestinal immune status against invading agents. Based on the current results, the gut's pH value was driven toward acidity in all algal treatments, but some failed to reach significance, as it was only a numerical difference. The acidic pH results due to the production of undissociated volatile fatty acids in the caeca (acetic, butyric, propionic, and lactic acids) and antimicrobial substances that efficiently eliminate or kill many different pathogens. The use of *Sargassum* sp. at 2%, *Spirulina* sp. at 5%, *Gracilaria* sp. at 0.5%, and 1% dramatically decreased the pH of the colon and increased intestinal acidity.

In some cases, the current increase in the blood indices may be related to the rich mineral content (Fe, Cu, and Zn) in the algal inclusions. Therefore, the higher inclusions of the algae showed varying results. The present results differ from another study where *Spirulina platensis* was used as a feed supplement in broiler chicks to examine hematological parameters, intestinal microbial population, and carcass traits. A total of 288 1-day-old broiler chicks were randomly assigned to one of the four groups, including control (basal diet with 0.04% zinc bacitracin) and birds getting the basal diet supplemented with 1% of *S. platensis* for the first 7 days (SP-7), for 21 days (SP-21), and for 35 days (SP-35). SP-35 had significantly lower levels of hemoglobin, erythrocytes, and hematocrit compared to other birds. SP-35 birds had a significantly lower number of leukocytes, lymphocytes, and eosinophils compared to the control (17). Al-Nasser et al. (3) fed Ross-308 male broilers five diets supplemented with different levels of *Scenedesmus* sp. microalgae and found that the WBC counts were higher in birds supplemented with microalgae as compared to those fed a basal diet. The current results showed that supplementing broilers with 1% of *Sargassum sp*. enhanced monocytes, compared to the other dietary groups. This reflects a higher innate response as monocytes have a role in both the inflammatory and anti-inflammatory responses related to the innate immune system. This is mainly due to the marine algae being a rich source of n-3 polyunsaturated fatty acids, carotenoids, B vitamins, and non-starch polysaccharides such as beta-glucans. These ingredients provide health benefits associated with the anti-inflammatory and antioxidant modulatory effects. The level of these effective ingredients vary among different species of algae (45).

## Conclusion

Supplementing broiler chickens with algae could enhance their cellular and humoral immune status and promote healthy microflora in their guts.

## Data Availability Statement

The raw data supporting the conclusions of this article will be made available by the authors, without undue reservation.

## Author Contributions

All authors listed have made a substantial, direct, and intellectual contribution to the work and approved it for publication.

## Conflict of Interest

The authors declare that the research was conducted in the absence of any commercial or financial relationships that could be construed as a potential conflict of interest.

## Publisher's Note

All claims expressed in this article are solely those of the authors and do not necessarily represent those of their affiliated organizations, or those of the publisher, the editors and the reviewers. Any product that may be evaluated in this article, or claim that may be made by its manufacturer, is not guaranteed or endorsed by the publisher.
