# Supplementary material for: Effects From Dietary Addition of Sargassum sp., Spirulina sp., or Gracilaria sp. Powder on Immune Status in Broiler Chickens
Source: Front Vet Sci. 2022 Jun 13;9:928235. doi: 10.3389/fvets.2022.928235 (PMC9234524; doi:10.3389/fvets.2022.928235)
Supplement: Supplementary file 1 [file Table_1.DOC]

**Table 1. Formulation and chemical analyses of the standard broiler diets, with no algal inclusion**

|  | **Broiler Starter** | **Broiler Grower** | **Broiler Finisher** |
| --- | --- | --- | --- |
| **Ingredients**  **(g/kg as fed)** |  |  |  |
| Corn | 491.02 | 553.30 | 598.65 |
| Soybean meal (SBM) | 426.55 | 370.53 | 319.78 |
| Soy Oil | 39.72 | 35.66 | 41.45 |
| Limestone | 11.76 | 11.5 | 10.55 |
| Salt | 3.145 | 2.67 | 2.49 |
| Soda Bicarbonate | 1.00 | 1.00 | 1.28 |
| Choline Chloride 60% | 0.68 | 0.81 | 0.76 |
| Di-Calcium Phosphate | 20.33 | 19.64 | 20.78 |
| L-Lysine 80% | 0.72 | 0.55 | 0.21 |
| DL-Methionine 98% | 3.0 | 2.25 | 2.043 |
| Premix Vit-Min-Additives* | 2.00 | 2.00 | 2.00 |
|  | **Broiler Starter** | **Broiler Grower** | **Broiler Finisher** |
| **Nutrient Levels** |  |  |  |
| Dry Matter (%) | 87.98 | 87.82 | 87.82 |
| Crude Protein (%) | 23.74 | 21.50 | 19.40 |
| Crude Fat (%) | 6.50 | 6.24 | 6.91 |
| Metabolizable Energy (kcal) | 3,000.00 | 3,050.00 | 3,143.00 |
| Crude ash (%) | 6.91 | 6.50 | 6.26 |
| Potassium (%) | 0.88 | 0.80 | 0.73 |
| Sodium (%) | 0.19 | 0.17 | 0.17 |
| Chlorine (%) | 0.24 | 0.21 | 0.20 |
| Total Calcium (%) | 0.98 | 0.95 | 0.93 |
| Available Phosphorus (%) | 0.46 | 0.44 | 0.46 |
| Digestible Lysine (%) | 1.26 | 1.12 | 0.97 |
| Digestible Methionine (%) | 0.63 | 0.52 | 0.48 |
| Digestible Methionine + Cystine (%) | 0.94 | 0.82 | 0.75 |
| Digestible Threonine (%) | 0.76 | 0.69 | 0.62 |
| Digestible Tryptophan (%) | 0.28 | 0.25 | 0.22 |
| Digestible Arginine (%) | 1.57 | 1.41 | 1.26 |
| Digestible Valine (%) | 0.98 | 0.89 | 0.80 |
| Digestible Isoleucine (%) | 0.97 | 0.87 | 0.78 |
| Digestible leucine (%) | 1.75 | 1.63 | 1.51 |
| Digestible Histidine (%) | 0.55 | 0.50 | 0.45 |
| Total Choline (mg/kg) | 1,850.00 | 1,800.00 | 1,650.00 |
| Na-Cl + K | 239.19 | 218.83 | 205.00 |

**^*^**Supplied per kg of premix: *trans*-retinol(A), 12500000IU; cholecalciferol(D3), 500000IU; α-tocopherol acetate(E), 75000 mg; thiamine (B1), 4500 mg; riboflavin (B2), 8000 mg; pyridoxine (B6), 5000 mg; vitamin B12, 22000 mg; pantothenic acid, 20000 mg; folic acid, 2000 mg; biotin, 200000 μg; Fe, 100000 mg; Co,250 mg; Mn, 100 mg; Cu, 10000 mg; Zn, 80000 mg; I, 1000 mg; Se, 300 mg; Mo, 0.5 mg; Ca, 7.7%; P, 0.01%; Na, 0.18%; Ash , 97%.

**Table 2. Formulation and chemical analyses of the broiler diet with 1% *Sargassum* sp.**

|  | **Broiler Starter** | | **Broiler Grower** | | **Broiler Finisher** |  |
| --- | --- | --- | --- | --- | --- | --- |
| **Ingredients (g/kg as fed)** |  | |  | |  |  |
| Corn | 477.33 | | 543.65 | | 588.71 |  |
| Soybean meal (SBM) | 424.54 | | 369.95 | | 319.25 |  |
| Soy Oil | 42.70 | | 37.73 | | 43.85 |  |
| Limestone | 12.78 | | 12.23 | | 11.20 |  |
| Salt | 1.55 | | 1.65 | | 1.66 |  |
| *Sargassum* sp. powder | 15.53 | | 10.00 | | 10.00 |  |
| Soda Bicarbonate | 1.00 | | 1.00 | | 1.00 |  |
| Choline Chloride 60% | 0.70 | | 0.82 | | 0.77 |  |
| Di-Calcium Phosphate | 18.02 | | 18.15 | | 19.29 |  |
| L-Lysine 80% | 0.75 | | 0.55 | | 0.22 |  |
| DL-Methionine 98% | 3.07 | | 2.256 | | 2.05 |  |
| Premix Vit-Min-Additives | 2.00 | | 2.00 | | 2.00 |  |
|  | **Broiler Starter** | | **Broiler Grower** | | **Broiler Finisher** |  |
| **Nutrient Levels** |  | |  | |  |  |
| Dry Matter (%) | 88.02 | | 87.85 | | 87.85 |  |
| Crude Protein (%) | 23.7 | | 21.50 | | 19.40 |  |
| Crude Fat (%) | 6.76 | | 6.42 | | 7.12 |  |
| Metabolizable Energy | 3,000.00 | | 3,050.00 | | 3,145.00 |  |
| Crude ash (%) | 6.98 | | 6.55 | | 6.30 |  |
| Potassium (%) | 0.93 | | 0.83 | | 0.76 |  |
| Sodium (%) | 0.19 | | 0.17 | | 0.17 |  |
| Chlorine (%) | 0.20 | | 0.19 | | 0.18 |  |
| Total Calcium (%) | 0.98 | | 0.95 | | 0.93 |  |
| Available Phosphorus (%) | 0.46 | | 0.44 | | 0.46 |  |
| Digestible Lysine (%) | 1.26 | | 1.12 | | 0.97 |  |
| Digestible Methionine (%) | 0.63 | | 0.52 | | 0.48 |  |
| Digestible Methionine + Cystine (%) | 0.94 | | 0.82 | | 0.75 |  |
| Digestible Threonine (%) | 0.76 | | 0.6 | | 0.62 |  |
| Threonine Tryptophan (%) | 0.28 | | 0.25 | | 0.22 |  |
| Digestible Arginine (%) | 1.56 | | 1.41 | | 1.26 |  |
| Digestible Valine (%) | 0.97 | | 0.8 | | 0.80 |  |
| Digestible Isoleucine (%) | 0.96 | | 0.87 | | 0.78 |  |
| Digestible leucine (%) | 1.75 | | 1.63 | | 1.50 |  |
| Digestible Histidine (%) | 0.55 | | 0.50 | | 0.45 |  |
| Total Choline (mg/kg) | 1,850.00 | | 1,800.00 | | 1,650.00 |  |
| Na-Cl + K | 263.00 | 234.55 | | 217.50 | |  |

**^*^**Supplied per kg of premix: *trans*-retinol(A), 12500000IU; cholecalciferol(D3), 500000IU; α-tocopherol acetate(E), 75000 mg; thiamine(B1), 4500 mg; riboflavin(B2), 8000 mg; pyridoxine(B6), 5000 mg; vitamin B12, 22000 mg; pantothenic acid, 20000 mg; folic acid, 2000 mg; biotin, 200000 μg; Fe, 100000 mg; Co,250 mg; Mn, 100 mg; Cu, 10000 mg; Zn, 80000 mg; I, 1000 mg; Se, 300 mg; Mo, 0.5 mg; Ca, 7.7%; P, 0.01%; Na, 0.18%; Ash , 97%.

**Table 3. Formulation and chemical analyses of the broiler diet with 2% *Sargassum* sp.**

|  | **Broiler Starter** | **Broiler Grower** | **Broiler Finisher** |
| --- | --- | --- | --- |
| **Ingredients (g/kg as fed)** |  |  |  |
| Corn | 470.25 | 533.23 | 578.20 |
| Soybean meal (SBM) | 424.39 | 369.50 | 318.81 |
| Soy Oil | 44.68 | 40.07 | 46.22 |
| Limestone | 13.07 | 12.88 | 11.86 |
| Salt | 1.10 | 1.00 | 1.056 |
| *Sargassum* sp. powder | 20.00 | 20.00 | 20.0 |
| Soda Bicarbonate | 1.00 | 1.00 | 1.00 |
| Choline Chloride 60% | 2.30 | 0.83 | 0.78 |
| Di-Calcium Phosphate | 17.3 | 16.66 | 17.80 |
| L-Lysine 80% | 0.7596 | 0.55 | 0.22 |
| DL-Methionine 98% | 3.08 | 2.26 | 2.05 |
| Premix Vit-Min-Additives | 2.00 | 2.00 | 2.00 |
|  | **Broiler Starter** | **Broiler Grower** | **Broiler Finisher** |
| **Nutrient Levels** |  |  |  |
| Dry Matter (%) | 88.063 | 87.89 | 87.89 |
| Crude Protein (%) | 23.69 | 21.50 | 19.40 |
| Crude Fat (%) | 6.94 | 6.63 | 7.33 |
| Metabolizable Energy | 3,000.00 | 3,050.00 | 3,145.000 |
| Crude ash (%) | 7.00 | 6.63 | 6.38 |
| Potassium (%) | 0.94 | 0.867 | 0.79 |
| Sodium (%) | 0.19 | 0.18 | 0.19 |
| Chlorine (%) | 0.21 | 0.18 | 0.18 |
| Total Calcium (%) | 0.98 | 0.95 | 0.93 |
| Available Phosphorus (%) | 0.46 | 0.45 | 0.46 |
| Digestible Lysine (%) (%) | 1.26 | 1.12 | 0.99 |
| Digestible Methionine (%) | 0.63 | 0.52 | 0.4 |
| Digestible Methionine + Cystine (%) | 0.94 | 0.82 | 0.75 |
| Digestible Threonine (%) | 0.76 | 0.69 | 0.62 |
| Threonine Tryptophan (%) | 0.28 | 0.25 | 0.22 |
| Digestible Arginine (%) | 1.56 | 1.41 | 1.26 |
| Digestible Valine (%) | 0.97 | 0.89 | 0.80 |
| Digestible Isoleucine (%) | 0.96 | 0.87 | 0.7 |
| Digestible leucine (%) | 1.74 | 1.62 | 1.50 |
| Digestible Histidine (%) | 0.55 | 0.50 | 0.45 |
| Total Choline (mg/kg) | 2,805.27 | 1,800.00 | 1,650.00 |
| Na-Cl + K | 263.00 | 250.30 | 233.25 |

**^*^**Supplied per kg of premix: *trans*-retinol(A), 12500000IU; cholecalciferol (D3), 500000IU; α-tocopherol acetate (E), 75000 mg; thiamine (B1), 4500 mg; riboflavin (B2), 8000 mg; pyridoxine(B6), 5000 mg; vitamin B12, 22000 mg; pantothenic acid, 20000 mg; folic acid, 2000 mg; biotin, 200000 μg; Fe, 100000 mg; Co,250 mg; Mn, 100 mg; Cu, 10000 mg; Zn, 80000 mg; I, 1000 mg; Se, 300 mg; Mo, 0.5 mg; Ca, 7.7%; P, 0.01%; Na, 0.18%; Ash , 97%.

**Table 4. Formulation and chemical analyses of the broiler diet with 5% *Spirulina* sp. inclusion**

|  | **Broiler Starter** | **Broiler Grower** | **Broiler Finisher** |
| --- | --- | --- | --- |
| **Ingredients (g/kg as fed)** |  |  |  |
| Corn | 571.32 | 598.35 | 632.45 |
| Soybean meal (SBM) | 292.75 | 276.41 | 245.172 |
| Soy Oil | 20.32 | 23.32 | 32.57 |
| Limestone | 13.73 | 13.19 | 11.83 |
| Salt | 1.00 | 1.00 | 1.00 |
| *Spirulina* sp. powder | 73.62 | 62.81 | 50.00 |
| Soda Bicarbonate | 1.00 | 1.00 | 2.23 |
| Choline Chloride 60% | 1.24 | 1.21 | 1.08 |
| Di-Calcium Phosphate | 17.37 | 17.06 | 18.73 |
| L-Lysine 80% | 2.81 | 1.75 | 1.17 |
| DL-Methionine 98% | 2.85 | 1.91 | 1.77 |
| Premix Vit-Min-Additives* | 2.00 | 2.00 | 2.00 |
|  | **Broiler Starter** | **Broiler Grower** | **Broiler Finisher** |
| **Nutrient Levels** |  |  |  |
| Dry Matter (%) | 87.71 | 87.67 | 87.72 |
| Crude Protein (%) | 22.92 | 21.50 | 19.40 |
| Crude Fat (%) | 4.81 | 5.16 | 6.14 |
| Metabolizable Energy (%) | 3,000.00 | 3,050.00 | 3,145.00 |
| Crude ash (%) | 6.43 | 6.19 | 6.08 |
| Potassium (%) | 0.76 | 0.73 | 0.67 |
| Sodium (%) | 0.20 | 0.18 | 0.20 |
| Chlorine (%) | 0.25 | 0.21 | 0.17 |
| Total Calcium (%) | 0.98 | 0.95 | 0.93 |
| Available Phosphorus (%) | 0.46 | 0.44 | 0.46 |
| Digestible Lysine (%) | 1.26 | 1.12 | 0.97 |
| Digestible Methionine (%) | 0.60 | 0.49 | 0.46 |
| Digestible Methionine + Cystine (%) | 0.94 | 0.82 | 0.75 |
| Digestible Threonine (%) | 0.76 | 0.71 | 0.64 |
| Threonine Tryptophan (%) | 0.21 | 0.20 | 0.18 |
| Digestible Arginine (%) | 1.44 | 1.35 | 1.21 |
| Digestible Valine (%) | 0.93 | 0.88 | 0.80 |
| Digestible Isoleucine (%) | 0.91 | 0.86 | 0.77 |
| Digestible leucine (%) | 1.71 | 1.63 | 1.51 |
| Digestible Histidine (%) | 0.47 | 0.46 | 0.42 |
| Total Choline (mg/kg) | 1,850.00 | 1,800.00 | 1,650.00 |
| Na-Cl + K | 210.25 | 205.00 | 205.00 |

**^*^**Supplied per kg of premix: *trans*-retinol(A), 12500000IU; cholecalciferol(D3), 500000IU; α-tocopherol acetate(E), 75000 mg; thiamine(B1), 4500 mg; riboflavin(B2), 8000 mg; pyridoxine(B6), 5000 mg; vitamin B12, 22000 mg; pantothenic acid, 20000 mg; folic acid, 2000 mg; biotin, 200000 μg; Fe, 100000 mg; Co,250 mg; Mn, 100 mg; Cu, 10000 mg; Zn, 80000 mg; I, 1000 mg; Se, 300 mg; Mo, 0.5 mg; Ca, 7.7%; P, 0.01%; Na, 0.18%; Ash , 97%.

**Table 5. Formulation and chemical analyses of the broiler diet with 7.5% *Spirulina* sp. inclusion**

|  | **Broiler Starter** | **Broiler Grower** | **Broiler Finisher** |
| --- | --- | --- | --- |
| **Ingredients (g/kg as fed)** |  |  |  |
| Corn | 569.65 | 602.85 | 611.56 |
| Soybean meal (SBM) | 292.92 | 261.21 | 241.93 |
| Soy Oil | 20.53 | 21.82 | 34.36 |
| Limestone | 13.75 | 13.48 | 12.35 |
| Salt | 1.00 | 1.00 | 1.00 |
| *Spirulina* sp. powder | 75.00 | 75.00 | 75.00 |
| Soda Bicarbonate | 1.00 | 1.09 | 1.091 |
| Choline Chloride 60% | 1.24 | 1.28 | 1.109 |
| Di-Calcium Phosphate | 17.31 | 16.55 | 17.61 |
| L-Lysine 80% | 2.77 | 1.89 | 0.64 |
| DL-Methionine 98% | 2.82 | 1.812 | 1.34 |
| Premix Vit-Min-Additives* | 2.00 | 2.00 | 2.00 |
|  | **Broiler Starter** | **Broiler Grower** | **Broiler Finisher** |
| **Nutrient Levels** |  |  |  |
| Dry Matter (%) | 87.715 | 87.66 | 87.70 |
| Crude Protein (%) | 23.00 | 21.60 | 20.62 |
| Crude Fat (%) | 4.8311 | 5.03 | 6.28 |
| Metabolizable Energy | 3,000.00 | 3,050.00 | 3,145.00 |
| Crude ash (%) | 6.43 | 6.18 | 6.06 |
| Potassium (%) | 0.76 | 0.72 | 0.69 |
| Sodium (%) | 0.200 | 0.20 | 0.20 |
| Chlorine (%) | 0.25 | 0.23 | 0.20 |
| Total Calcium (%) | 0.98 | 0.95 | 0.93 |
| Available Phosphorus (%) | 0.46 | 0.44 | 0.46 |
| Digestible Lysine (%) | 1.26 | 1.17 | 0.97 |
| Digestible Methionine | 0.600 | 0.49 | 0.43 |
| Digestible Methionine + Cystine (%) | 0.94 | 0.82 | 0.75 |
| Digestible Threonine (%) | 0.76 | 0.72 | 0.69 |
| Threonine Tryptophan (%) | 0.21 | 0.19 | 0.18 |
| Digestible Arginine (%) | 1.44 | 1.35 | 1.29 |
| Digestible Valine (%) | 0.94 | 0.89 | 0.85 |
| Digestible Isoleucine (%) | 0.92 | 0.86 | 0.82 |
| Digestible leucine (%) | 1.71 | 1.64 | 1.59 |
| Digestible Histidine (%) | 0.48 | 0.45 | 0.43 |
| Total Choline (mg/kg) | 1,850.00 | 1,800.00 | 1,650.00 |
| Na-Cl + K | 211.158 | 205.00 | 205.00 |

**^*^**Supplied per kg of premix: *trans*-retinol(A), 12500000IU; cholecalciferol(D3), 500000IU; α-tocopherol acetate(E), 75000 mg; thiamine(B1), 4500 mg; riboflavin(B2), 8000 mg; pyridoxine(B6), 5000 mg; vitamin B12, 22000 mg; pantothenic acid, 20000 mg; folic acid, 2000 mg; biotin, 200000 μg; Fe, 100000 mg; Co,250 mg; Mn, 100 mg; Cu, 10000 mg; Zn, 80000 mg; I, 1000 mg; Se, 300 mg; Mo, 0.5 mg; Ca, 7.7%; P, 0.01%; Na, 0.18%; Ash , 97%.

**Table 6. Formulation and chemical analyses of the broiler diet with 0.5% *Gracilaria* sp. inclusion**

|  | **Broiler Starter** | **Broiler Grower** | **Broiler Finisher** |
| --- | --- | --- | --- |
| **Ingredients (g/kg as fed)** |  |  |  |
| Corn | 487.14 | 549.59 | 594.94 |
| Soybean meal (SBM) | 425.53 | 370.00 | 319.36 |
| Soy Oil | 40.78 | 36.64 | 42.68 |
| Limestone | 12.78 | 12.44 | 11.33 |
| Salt | 1.63 | 1.38 | 1.57 |
| *Gracilaria* sp. powder | 6.54 | 5.57 | 5.00 |
| Soda Bicarbonate | 1.00 | 1.00 | 1.00 |
| Choline Chloride 60% | 0.69 | 0.814 | 0.76 |
| Di-Calcium Phosphate | 18.12 | 17.75 | 19.09 |
| L-Lysine 80% | 0.73 | 0.55 | 0.22 |
| DL-Methionine 98% | 3.06 | 2.25 | 2.04 |
| Premix Vit-Min-Additives | 2.00 | 2.00 | 2.00 |
|  | **Broiler Starter** | **Broiler Grower** | **Broiler Finisher** |
| **Nutrient Levels** |  |  |  |
| Dry Matter (%) | 87.97 | 87.82 | 87.83 |
| Crude Protein (%) | 23.73 | 21.50 | 19.40 |
| Crude Fat (%) | 6.60 | 6.33 | 7.02 |
| Metabolizable Energy Poultry | 3,000.00 | 3,050.00 | 3,145.00 |
| Crude ash (%) | 7.00 | 6.57 | 6.32 |
| Potassium (%) | 0.93 | 0.84 | 0.77 |
| Sodium (%) | 0.19 | 0.17 | 0.17 |
| Chlorine (%) | 0.20 | 0.18 | 0.18 |
| Total Calcium (%) | 0.98 | 0.95 | 0.93 |
| Available Phosphorus (%) | 0.46 | 0.44 | 0.46 |
| Digestible Lysine (%) | 1.26 | 1.12 | 0.97 |
| Digestible Methionine | 0.63 | 0.530 | 0.48 |
| Digestible Methionine + Cystine (%) | 0.94 | 0.82 | 0.75 |
| Digestible Threonine (%) | 0.76 | 0.69 | 0.62 |
| Threonine Tryptophan (%) | 0.28 | 0.25 | 0.22 |
| Digestible Arginine (%) | 1.57 | 1.41 | 1.26 |
| Digestible Valine (%) | 0.97 | 0.89 | 0.8 |
| Digestible Isoleucine (%) | 0.97 | 0.87 | 0.78 |
| Digestible leucine (%) | 1.75 | 1.63 | 1.51 |
| Digestible Histidine (%) | 0.55 | 0.50 | 0.46 |
| Total Choline (mg/kg) | 1,850.00 | 1,800.00 | 1,650.00 |
| Na-Cl + K | 263.00 | 239.29 | 220.15 |

**\**

**^*^**Supplied per kg of premix: *trans*-retinol(A), 12500000IU; cholecalciferol(D3), 500000IU; α-tocopherol acetate(E), 75000 mg; thiamine(B1), 4500 mg; riboflavin(B2), 8000 mg; pyridoxine(B6), 5000 mg; vitamin B12, 22000 mg; pantothenic acid, 20000 mg; folic acid, 2000 mg; biotin, 200000 μg; Fe, 100000 mg; Co,250 mg; Mn, 100 mg; Cu, 10000 mg; Zn, 80000 mg; I, 1000 mg; Se, 300 mg; Mo, 0.5 mg; Ca, 7.7%; P, 0.01%; Na, 0.18%; Ash , 97%.

**Table 7. Formulation and chemical analyses of the broiler diet with 1% *Gracilaria* sp.**

|  | **Broiler Starter** | **Broiler Grower** | **Broiler Finisher** |
| --- | --- | --- | --- |
| **Ingredients (g/kg as fed)** |  |  |  |
| Corn | 478.97 | 545.33 | 590.41 |
| Soybean meal (SBM) | 425.82 | 369.79 | 319.08 |
| Soy Oil | 43.51 | 37.87 | 43.98 |
| Limestone | 13.30 | 13.12 | 12.10 |
| Salt | 1.00 | 1.00 | 1.00 |
| *Gracilaria* sp. powder | 10.00 | 10.00 | 10.00 |
| Soda Bicarbonate | 1.00 | 1.00 | 1.00 |
| Choline Chloride 60% | 3.62 | 0.82 | 0.77 |
| Di-Calcium Phosphate | 16.97 | 16.26 | 17.40 |
| L-Lysine 80% | 0.73 | 0.55 | 0.22 |
| DL-Methionine 98% | 3.07 | 2.25 | 2.04 |
| Premix Vit-Min-Additives | 2.00 | 2.00 | 2.00 |
|  | **Broiler Starter** | **Broiler Grower** | **Broiler Finisher** |
| **Nutrient Levels** |  |  |  |
| Dry Matter (%) | 88.04 | 87.83 | 87.84 |
| Crude Protein (%) | 23.72 | 21.50 | 19.40 |
| Crude Fat (%) | 6.84 | 6.44 | 7.14 |
| Metabolizable Energy Poultry | 3,000.00 | 3,050.00 | 3,145.00 |
| Crude ash (%) | 7.05 | 6.69 | 6.44 |
| Potassium (%) | 0.95 | 0.88 | 0.80 |
| Sodium (%) | 0.20 | 0.19 | 0.19 |
| Chlorine (%) | 0.24 | 0.19 | 0.18 |
| Total Calcium (%) | 0.98 | 0.95 | 0.93 |
| Available Phosphorus (%) | 0.46 | 0.44 | 0.46 |
| Digestible Lysine (%) | 1.26 | 1.12 | 0.97 |
| Digestible Methionine (%) | 0.63 | 0.52 | 0.48 |
| Digestible Methionine + Cystine (%) | 0.94 | 0.82 | 0.75 |
| Digestible Threonine (%) | 0.76 | 0.69 | 0.62 |
| Threonine Tryptophan (%) | 0.28 | 0.25 | 0.22 |
| Digestible Arginine (%) | 1.57 | 1.41 | 1.26 |
| Digestible Valine (%) | 0.97 | 0.89 | 0.80 |
| Digestible Isoleucine (%) | 0.97 | 0.87 | 0.78 |
| Digestible leucine (%) | 1.75 | 1.63 | 1.50 |
| Digestible Histidine (%) | 0.55 | 0.50 | 0.45 |
| Total Choline (mg/kg) | 3,606.53 | 1,800.00 | 1,650.00 |
| Na-Cl + K | 263.00 | 255.62 | 238.57 |

**^*^**Supplied per kg of premix: *trans*-retinol(A), 12500000IU; cholecalciferol(D3), 500000IU; α-tocopherol acetate(E), 75000 mg; thiamine(B1), 4500 mg; riboflavin(B2), 8000 mg; pyridoxine(B6), 5000 mg; vitamin B12, 22000 mg; pantothenic acid, 20000 mg; folic acid, 2000 mg; biotin, 200000 μg; Fe, 100000 mg; Co,250 mg; Mn, 100 mg; Cu, 10000 mg; Zn, 80000 mg; I, 1000 mg; Se, 300 mg; Mo, 0.5 mg; Ca, 7.7%; P, 0.01%; Na, 0.18%; Ash , 97%.
